# The vicious cycle: hyperventilation-induced alkalosis mimicking refractory hypocalcaemia after thyroidectomy

**DOI:** 10.1093/jscr/rjag327

**Published:** 2026-04-28

**Authors:** Louis Britten-Jones, Nicholas Shannon, Bruce Ashford

**Affiliations:** Department of General Surgery, Wollongong Hospital, Illawarra Shoalhaven Local Health District, Wollongong, NSW 2522, Australia; Department of General Surgery, Wollongong Hospital, Illawarra Shoalhaven Local Health District, Wollongong, NSW 2522, Australia; Department of General Surgery, Wollongong Hospital, Illawarra Shoalhaven Local Health District, Wollongong, NSW 2522, Australia; Faculty of Science, Medicine and Health, Graduate School of Medicine, University of Wollongong, Wollongong, NSW 2500, Australia

**Keywords:** postoperative hypoparathyroidism, respiratory alkalosis, ionized calcium, total thyroidectomy, calcium supplementation

## Abstract

Postoperative hypoparathyroidism is the most common complication following total thyroidectomy and is usually transient. Calcium supplementation is guided by serial biochemical monitoring, targeting low-normal serum calcium to avoid hypocalcaemia while facilitating parathyroid recovery. Respiratory alkalosis reduces ionized calcium, though its impact postoperatively is under-recognized. We report a 30-year-old male who developed hypoparathyroidism after total thyroidectomy and neck dissection for medullary thyroid carcinoma. Anxiety-driven hyperventilation caused recurrent respiratory alkalosis, producing symptomatic reductions in ionized calcium despite escalating intravenous replacement. This culminated in a code blue activation, ICU admission, and subsequent hypercalcaemia. We propose that respiratory alkalosis contributed to apparent refractory hypocalcaemia, and that aggressive calcium replacement may have delayed parathyroid recovery through iatrogenic suppression. This case highlights the importance of recognizing respiratory alkalosis as a reversible contributor to hypocalcaemia and exercising caution with calcium supplementation in postoperative hypoparathyroidism.

## Introduction

Postoperative hypoparathyroidism causes hypocalcaemia and is the most common complication of total thyroidectomy [1]. It is typically transient. Treatment requires calcium supplementation guided by serial biochemical monitoring. Aiming for low-normal serum calcium avoids hypocalcaemia while stimulating parathyroid recovery [2, 3]. Under normal physiology, calcium-sensing receptors on parathyroid glands detect hypocalcaemia and stimulate parathyroid hormone production to restore normocalcaemia. Alkalaemia is known to decrease ionized calcium (iCa) levels, as albumin increasingly binds calcium when hydrogen ion concentration falls [4]. We report a case of postoperative hypoparathyroidism in which persistent, anxiety-driven respiratory alkalosis led to inappropriately aggressive calcium replacement, potentially delaying parathyroid recovery and resulting in ICU admission and hypercalcaemia.

## Case report

A 30-year-old male presented for surgical management of a fine needle aspiration proven medullary thyroid carcinoma (MTC). His medical history included depression, anxiety, cannabis use disorder, opioid use disorder and Arnold-Chiari malformation. A thyroid nodule measuring 9 × 6 mm was an incidental finding on upper limb arterial Doppler ultrasound ordered to investigate bilateral hand paraesthesias. Repeat ultrasound 6 months later demonstrated an 18 x 16 mm nodule with paratracheal and level IV lymphadenopathy. Fine needle aspiration cytology was Bethesda category VI and favoured medullary thyroid carcinoma, with positive calcitonin staining and amyloid deposition. Preoperative calcitonin was 191 ng/L (normal range < 12 ng/L) and carcinoembryonic antigen (CEA) was 6 μg/L (normal range < 2.5 μg/L).

A total thyroidectomy was performed with a right-sided paratracheal and level III-IV neck dissection. The left superior, left inferior and right superior parathyroid glands were identified and preserved. The right inferior parathyroid gland was dissected and reimplanted to the sternocleidomastoid muscle.

Initial management was ward based, with routine postoperative biochemistry demonstrating hypoparathyroidism. Parathyroid hormone (PTH) was <0.6 pmol/L (normal range 1.0–7.0 pmol/L) and oral calcium replacement was commenced.

Overnight, urgent medical review was sought with concern for symptomatic hypocalcaemia. The patient was distressed and tachycardic with carpopaedal spasm, muscle fasciculations, subjective loss of motor control and positive Trousseau’s sign. Heart rate was 119 beats per minute, blood pressure was 119/74 mm Hg, respiratory rate was 18 breaths per minute, SpO_2_ was 97% and temperature was 36.2°C. ECG showed sinus tachycardia with no QTc prolongation. Venous blood gas (VBG) demonstrated hypocalcaemia with iCa 1.05 mmol/L (normal range 1.15–1.30 mmol/L), pH 7.39 and PCO_2_ 43.9 mm Hg. Corrected calcium was 2.09 mmol/L (normal range 2.10–2.60 mmol/L) and magnesium was 0.83 mmol/L (normal range 0.70–1.10 mmol/L). Other routine postoperative biochemistry was unremarkable. Intravenous calcium gluconate 2.2 mmol was administered.

On postoperative Day 1, the patient developed recurrent symptoms that appeared disproportionate to the degree of biochemical hypocalcaemia. VBG demonstrated hypocalcaemia with iCa 1.06 mmol/L, pH 7.43, and PCO_2_ 40.2 mm Hg. Intravenous calcium gluconate 2.2 mmol was again administered. Later that day, symptoms recurred and VBG showed hypocalcaemia and a respiratory alkalosis, with iCa 1.02 mmol/L, pH 7.45 and PCO_2_ 37.7 mm Hg. Magnesium was 0.78 mmol/L. Intravenous calcium gluconate 2.2 mmol and magnesium 20 mmol was administered. The endocrinology team reviewed the patient and noted that although the patient had hypoparathyroidism, the patient’s symptoms were more significant than would be expected for their calcium levels.

A code blue was activated with concern for impending respiratory failure on postoperative Day 2. The patient was found to have facial swelling with new hoarseness, perioral paraesthesia, carpopaedal spasm, and positive Trousseau’s sign. Heart rate was 130 beats per minute, blood pressure was 153/88 mm Hg and respiratory rate was 36 breaths per minute. Drain output was 150 ml since the operation. The patient was profoundly diaphoretic with increased work of breathing and was commenced on a 15 L/min oxygen via non-rebreather mask. There were no rashes and no exposure to new medication or food. Flexible nasendoscopy was performed, finding normal vocal cord movement and no oedema. VBG showed iCa 0.99 mmol/L, pH 7.38 and PCO_2_ 43.5 mm Hg. ECG showed normal QTc of 422 ms. A cumulative 6.6 mmol of intravenous calcium gluconate was given. Despite the reassurances of the surgical team, the patient was transferred to ICU for closer monitoring of calcium levels. Following transfer to ICU, arterial blood gas (ABG) demonstrated normocalcaemia and significant respiratory alkalosis, with iCa 1.29 mmol/L, pH 7.52, and PaCO_2_ 24.9 mm Hg. Later that day, ABG again showed hypocalcaemia alongside respiratory alkalosis, with iCa 1.04 mmol/L, pH 7.48, and PaCO_2_ 31.0 mm Hg. Ongoing anxiety was noted. A calcium gluconate infusion was commenced and titrated to 50 mL/hr.

Hypocalcaemia, anxiety and hyperventilation continued while the patient remained in ICU, with management primarily focused on calcium escalation. On postoperative Day 3, hypocalcaemia with respiratory alkalosis was present on ABG, with iCa 0.99 mmol/L, pH 7.49, and PaCO_2_ 30.3 mm Hg. Ongoing anxiety and paraesthesias that morning were noted. The rate of the calcium gluconate infusion was increased to 100 ml/hr. Drug and alcohol review diagnosed acute cannabis withdrawal and mild opioid withdrawal and noted that this was likely contributing to the patient’s symptomatology. Benzodiazepine therapy was recommended for withdrawal symptoms. The endocrinology team provided ongoing advice regarding calcium replacement.

An unsuccessful attempt was made to cease the calcium infusion on postoperative day four. Anxiety related to the cessation of the infusion was noted. Repeat ABG following restart of infusion again demonstrated respiratory alkalosis, with pH 7.48 and PaCO_2_ 33.4 mm Hg. iCa was 1.22 mmol/L. In order to maintain normocalcaemia following cessation of infusion, a normal-high serum corrected calcium (CorrCa) of 2.5–2.6 mmol/L was targeted.

Upon stepdown to the ward, the patient was found to be hypercalcaemic with CorrCa 2.79 mmol/L. The patient was subsequently discharged on oral calcium supplementation.

Following discharge, the patient represented to the emergency department with generalized paraesthesias and palpitations, resembling episodes of hypocalcaemia. VBG demonstrated iCa 1.14 alongside respiratory alkalosis. Symptoms were attributed to alcohol withdrawal in the setting of significant alcohol intake the previous evening.

Upon review by the surgical team 1 month later, the patient was normocalcaemic and oral calcium supplementation was ceased. The patient also followed up with endocrinology and was referred for genetic counselling and testing for RET mutations associated with MEN2. Genetic testing showed no germline abnormality, supporting a diagnosis of sporadic MTC. A referral to a psychologist for ongoing stress was also made. Drug and alcohol follow up noted that the patient had ceased cannabis use.

## Discussion

This case illustrates how postoperative hypoparathyroidism can become diagnostically and therapeutically complex when concurrent respiratory alkalosis lowers ionized calcium and amplifies symptoms. Transient hypoparathyroidism is the most common complication following total thyroidectomy, with an estimated prevalence of 19%–38% [5]. This occurs due to partial revascularization and surgical trauma to the parathyroid glands. Functional recovery occurs as blood supply and cellular activity is restored [6]. Treatment of hypoparathyroidism involves calcium and vitamin D supplementation, but there is a lack of high-quality evidence regarding the extent and duration of supplementation [7]. Guidelines suggest replacement with an aim for low-normal serum calcium, avoiding hypocalcaemia and its associated risks while also enabling parathyroid recovery by avoiding iatrogenic parathyroid suppression [2, 3].

In addition to postoperative hypoparathyroidism, another important mechanism of symptomatic hypocalcaemia is respiratory alkalosis. As hydrogen ion concentration falls, calcium ions will increasingly bind to albumin, transiently decreasing ionized calcium levels, while total serum calcium remains constant [4, 8]. Hyperventilation syndrome is an under-recognized condition featuring inappropriate alveolar hyperventilation leading to respiratory alkalosis, often driven by anxiety [9].

In this case, early postoperative hypoparathyroidism was demonstrated, but the severity and recurrence of symptoms appeared disproportionate to the biochemical abnormality alone. Ongoing symptoms despite calcium supplementation prompted escalation to high-dose intravenous calcium, at rates of up to 2.2 mmol/hr of calcium gluconate, without sustained improvement in iCa. The patient was also transferred to a high-acuity setting for closer monitoring.

We suggest that the reason for persistently low iCa despite intravenous calcium supplementation is two-fold. Firstly, arterial and venous blood gas readings throughout the patient’s admission demonstrated respiratory alkalosis, which is associated with a transient hypocalcaemia [4, 8]. This was likely due to hyperventilation secondary to anxiety. The patient’s background history of anxiety was likely exacerbated by acute cannabis withdrawal, the activation of a code blue and an urgent transfer to ICU. Furthermore, we posit that the early symptoms of hypocalcaemia may have precipitated anxiety and hyperventilation, creating a vicious cycle where ionized calcium levels decreased further and symptoms became more pronounced.

Secondly, aggressive calcium replacement and allowance of a permissive hypercalcaemia may have delayed parathyroid recovery by iatrogenically suppressing parathyroid activity. Under normal physiology, parathyroid glands detect changes in ionised calcium levels via the calcium-sensing receptor (CaSR). When CaSR detects normal or high calcium, PTH secretion is inhibited via a negative feedback loop [10]. The stimulation theory of calcium supplementation states that overly aggressive calcium replacement will suppress parathyroid function and delay the recovery of stunned parathyroid glands [11, 12]. Guidelines now recommend aiming for low-normal calcium levels when replacing calcium in patients with hypoparathyroidism [2, 3].

This case also highlights the importance of multidisciplinary assessment when postoperative symptoms are disproportionate to the apparent biochemical abnormality. In this patient, earlier multidisciplinary input involving endocrinology, drug and alcohol, and mental health services may have identified reversible contributors to anxiety, hyperventilation, and recurrent respiratory alkalosis, which complicated interpretation of the patient’s calcium status. This is particularly relevant in patients at risk of substance withdrawal or significant perioperative anxiety.

Additionally, this case underscores the complexity of calcium replacement in patients with postoperative hypoparathyroidism. Potential parathyroid suppression from over-replacement, as well as multifactorial contributors to symptomatic hypocalcaemia, should be considered when planning calcium replacement. Replacement should aim for low-normal calcium levels to support parathyroid recovery. Endocrine surgeons and all those involved in the care of thyroidectomy patients must have a strong understanding of these factors.

In this patient with postoperative hypoparathyroidism, aggressive calcium replacement in the setting of respiratory alkalaemia contributed to systemic hypercalcaemia and may have contributed to escalation of care. While anxiety is exceedingly common in the perioperative period [13], and hypoparathyroidism is the most common complication of thyroid surgery [5], the impact of anxiety on calcium replacement for postoperative hypoparathyroidism has not yet been discussed. Surgeons must be aware of this issue to guide judicious calcium supplementation and enable early parathyroid recovery.
